# P-2307. Premature Discontinuation of Trimethoprim-Sulfamethoxazole Prophylaxis in Abdominal Transplant Recipients: A Deeper Dive

**DOI:** 10.1093/ofid/ofae631.2459

**Published:** 2025-01-29

**Authors:** Tyler T Tinkham, Dan Ilges, Madeline R Hudson, Cassandra D Votruba, Rebecca Corey, McKenna J Beemiller, Justin M Potter, Alan Gonzalez, Ashkan Rastegar, Blanca Lizaola-Mayo, Lavanya Kodali, Holenarasipur R Vikram

**Affiliations:** Mayo Clinic, Phoenix, Arizona; Mayo Clinic Arizona, Phoenix, Arizona; Mayo Clinic, Phoenix, Arizona; Mayo Clinic Arizona, Phoenix, Arizona; Mayo Clinic, Phoenix, Arizona; Mayo Clinic, Phoenix, Arizona; Mayo Clinic Hospital, Phoenix, Arizona; Mayo Clinic, Phoenix, Arizona; Mayo Clinic School of Health Sciences, Phoenix, Arizona; Mayo Clinic/ Transplant Center, Phoenix, Arizona; Mayo Clinic, Phoenix, Arizona; Mayo Clinic, Phoenix, Arizona

## Abstract

**Background:**

Trimethoprim-sulfamethoxazole (TS) prophylaxis can prevent Pneumocystis jirovecii (PCP) and other opportunistic infections (OIs), such as intestinal protozoa, Toxoplasma, Listeria, and Nocardia. We sought to assess the frequency, causative factors, and impact of early TS discontinuation in abdominal SOT recipients.

**Methods:**

This is a single-center, retrospective cohort study of adult abdominal SOT recipients at Mayo Clinic Arizona between 1/1/2021 and 6/30/2023. Objectives included the rate and reasons behind early TS discontinuation and whether TS prophylaxis was reinitiated. Early discontinuation was defined as cessation of TS prophylaxis within 6 months post-transplant for ≥7 days. Secondary outcomes included median duration of therapy, alternative prophylactic agent utilized, and incidence of TS preventable OI. Patients were excluded if they had no TS ordered, died <180 days from transplant, or received a thoracic organ transplant.

**Results:**

942 abdominal SOT recipients were identified. Thus far 171 liver, 11 Kidney, and 29 liver/kidney have been reviewed and included in the current analysis. TS was discontinued early in 60 (28%) patients. Predominant reasons for discontinuation were hyperkalemia (45%), cytopenias (17%), and acute kidney injury (15%). Allergy was the indication for discontinuation in 1 patient. Median duration of TS before discontinuation was 39 days (IQR 66). TS was not resumed in 78% of cases. The predominant reason for non-resumption was alternative prophylaxis with no clear intent to rechallenge TS (70%). The main discontinuation reason had resolved in 70% that stopped due to cytopenias, 67% that stopped due to liver injury, and 96% that stopped due to hyperkalemia. Alternative prophylaxis included pentamidine (50%), dapsone (32%), atovaquone (5%), none (13%). No patients developed PCP or other TS preventable OI during 9-month follow-up post-SOT.

**Conclusion:**

TS is discontinued prematurely in a significant number of SOT recipients and is often not resumed despite resolution of the offending cause. Given its proven track record in preventing several OIs in SOT recipients, providers should document reason(s) for TS discontinuation and consider resumption of TS after reasons for discontinuation resolve.

**Disclosures:**

All Authors: No reported disclosures

